# Supplementary material for: P-TRAP: a Panicle Trait Phenotyping tool
Source: BMC Plant Biol. 2013 Aug 29;13:122. doi: 10.1186/1471-2229-13-122 (PMC3848748; doi:10.1186/1471-2229-13-122)
Supplement: Additional file 7 — P-TRAP source code. The Netbeans project that contains the source code of the application. [file 1471-2229-13-122-S7.zip › javadoc.zip!/javadoc/org-basmati-workspace/org/basmati/workspace/VeiwParticlesAnchors.html]

VeiwParticlesAnchors (WorkSpace)


JavaScript is disabled on your browser.


- Package
- Class
- Tree
- Deprecated
- Index
- Help

*org.basmati.workspace 1.0*

- Prev Class
- Next Class

- Frames
- No Frames

- All Classes

- Summary:
- Nested |
- Field |
- Constr |
- Method

- Detail:
- Field |
- Constr |
- Method


org.basmati.workspace

## Class VeiwParticlesAnchors

- java.lang.Object
- - org.openide.util.SharedClassObject
  - - org.openide.util.actions.SystemAction
    - - org.openide.util.actions.BooleanStateAction
      - - org.basmati.workspace.VeiwParticlesAnchors

- All Implemented Interfaces:
  :   java.awt.event.ActionListener, java.io.Externalizable, java.io.Serializable, java.util.EventListener, javax.swing.Action, org.openide.util.actions.Presenter, org.openide.util.actions.Presenter.Menu, org.openide.util.actions.Presenter.Popup, org.openide.util.actions.Presenter.Toolbar, org.openide.util.HelpCtx.Provider

  ---

    

  ```
  public final class VeiwParticlesAnchors
  extends org.openide.util.actions.BooleanStateAction
  implements java.awt.event.ActionListener
  ```

  The handler of toggling the anchor points of the grains ON and OFF.

  Author:
  :   faroqal-tam

  See Also:
  :   Serialized Form

- - ### Nested Class Summary

    - ### Nested classes/interfaces inherited from interface org.openide.util.actions.Presenter

      `org.openide.util.actions.Presenter.Menu, org.openide.util.actions.Presenter.Popup, org.openide.util.actions.Presenter.Toolbar`
  - ### Field Summary

    - ### Fields inherited from class org.openide.util.actions.BooleanStateAction

      `PROP_BOOLEAN_STATE`
    - ### Fields inherited from class org.openide.util.actions.SystemAction

      `PROP_ENABLED, PROP_ICON`
    - ### Fields inherited from interface javax.swing.Action

      `ACCELERATOR_KEY, ACTION_COMMAND_KEY, DEFAULT, DISPLAYED_MNEMONIC_INDEX_KEY, LARGE_ICON_KEY, LONG_DESCRIPTION, MNEMONIC_KEY, NAME, SELECTED_KEY, SHORT_DESCRIPTION, SMALL_ICON`
  - ### Constructor Summary

    Constructors

    Constructor and Description

    `VeiwParticlesAnchors()`
  - ### Method Summary

    Methods

    | Modifier and Type | Method and Description |
    | --- | --- |
    | `void` | `actionPerformed(java.awt.event.ActionEvent event)` |
    | `org.openide.util.HelpCtx` | `getHelpCtx()` |
    | `javax.swing.JMenuItem` | `getMenuPresenter()` |
    | `java.lang.String` | `getName()` |
    | `java.awt.Component` | `getToolbarPresenter()` |
    | `protected java.lang.String` | `iconResource()` |

    - ### Methods inherited from class org.openide.util.actions.BooleanStateAction

      `getBooleanState, getPopupPresenter, initialize, setBooleanState`
    - ### Methods inherited from class org.openide.util.actions.SystemAction

      `clearSharedData, createPopupMenu, createToolbarPresenter, get, getIcon, getIcon, getValue, isEnabled, linkActions, putValue, setEnabled, setIcon`
    - ### Methods inherited from class org.openide.util.SharedClassObject

      `addNotify, addPropertyChangeListener, equals, finalize, findObject, findObject, firePropertyChange, getLock, getProperty, hashCode, putProperty, putProperty, readExternal, removeNotify, removePropertyChangeListener, reset, writeExternal, writeReplace`
    - ### Methods inherited from class java.lang.Object

      `clone, getClass, notify, notifyAll, toString, wait, wait, wait`
    - ### Methods inherited from interface javax.swing.Action

      `addPropertyChangeListener, removePropertyChangeListener`

- - ### Constructor Detail


    - #### VeiwParticlesAnchors

      ```
      public VeiwParticlesAnchors()
      ```
  - ### Method Detail


    - #### getName

      ```
      public java.lang.String getName()
      ```

      **Specified by:**
      :   `getName` in class `org.openide.util.actions.SystemAction`


    - #### actionPerformed

      ```
      public void actionPerformed(java.awt.event.ActionEvent event)
      ```

      **Specified by:**
      :   `actionPerformed` in interface `java.awt.event.ActionListener`

      **Overrides:**
      :   `actionPerformed` in class `org.openide.util.actions.BooleanStateAction`


    - #### getHelpCtx

      ```
      public org.openide.util.HelpCtx getHelpCtx()
      ```

      **Specified by:**
      :   `getHelpCtx` in interface `org.openide.util.HelpCtx.Provider`

      **Specified by:**
      :   `getHelpCtx` in class `org.openide.util.actions.SystemAction`


    - #### getToolbarPresenter

      ```
      public java.awt.Component getToolbarPresenter()
      ```

      **Specified by:**
      :   `getToolbarPresenter` in interface `org.openide.util.actions.Presenter.Toolbar`

      **Overrides:**
      :   `getToolbarPresenter` in class `org.openide.util.actions.BooleanStateAction`


    - #### getMenuPresenter

      ```
      public javax.swing.JMenuItem getMenuPresenter()
      ```

      **Specified by:**
      :   `getMenuPresenter` in interface `org.openide.util.actions.Presenter.Menu`

      **Overrides:**
      :   `getMenuPresenter` in class `org.openide.util.actions.BooleanStateAction`


    - #### iconResource

      ```
      protected java.lang.String iconResource()
      ```

      **Overrides:**
      :   `iconResource` in class `org.openide.util.actions.SystemAction`


- Package
- Class
- Tree
- Deprecated
- Index
- Help

*org.basmati.workspace 1.0*

- Prev Class
- Next Class

- Frames
- No Frames

- All Classes

- Summary:
- Nested |
- Field |
- Constr |
- Method

- Detail:
- Field |
- Constr |
- Method
